# Developmental Hip Dysplasia: An Epidemiological Nationwide Study in Italy from 2001 to 2016

**DOI:** 10.3390/ijerph18126589

**Published:** 2021-06-18

**Authors:** Umile Giuseppe Longo, Rocco Papalia, Sergio De Salvatore, Laura Ruzzini, Ilaria Piergentili, Leonardo Oggiano, Pier Francesco Costici, Vincenzo Denaro

**Affiliations:** 1 Department of Orthopedic and Trauma Surgery, Campus Bio-Medico University of Rome, 00128 Rome, Italy; r.papalia@unicampus.it (R.P.); s.desalvatore@unicampus.it (S.D.S.); ilaria.piergentili94@gmail.com (I.P.); denaro@unicampus.it (V.D.); 2 Orthopedic Unit, Department of Surgery, Bambino Gesù Children’s Hospital, 00165 Rome, Italy; laura.ruzzini@opbg.net (L.R.); leonardo.oggiano@opbg.net (L.O.); pierfrancesco.costici@opbg.net (P.F.C.)

**Keywords:** developmental dysplasia of the hip, DDH, national hospital discharge reports, NHDR, SDO, Italian

## Abstract

Developmental Dysplasia of the Hip (DDH) includes a broad spectrum of hip abnormalities. DDH requires early diagnosis and treatment; however, no international consensus on screening protocol and treatment is provided in the literature. Epidemiological studies are helpful to understand the national variation of a specific surgical procedure and compare it with that of other countries. Data provided by different countries could allow researchers to provide international guidelines for DDH screening and treatment. Limited data are reported regarding trends of hospitalization for DDH, and no public database is available. The purpose of this study was to estimate annual admissions for DDH in Italian patients from 2001 to 2016, based on the hospitalization reports. Data of this study were collected from the National Hospital Discharge Reports (SDO) reported at the Italian Ministry of Health. Descriptive statistical analyses were performed. From 2001 to 2016, 3103 hospitalizations for DDH were recorded in Italy, with a mean incidence of 2.33 (per 100,000 young inhabitants). Females of the 0–4 years old group represented the majority of patients hospitalized for DDH.

## 1. Introduction

Developmental dysplasia of the hip (DDH) includes a wide range of hip alterations (from simple dysplasia to dislocation). DDH is characterized by pathological modification of the acetabular cup and/or femoral head, with consequent soft tissue abnormalities (hip capsule and ligaments). The femoral head could be within the acetabular cup (located), partially out of the acetabular cup (subluxated) or outside of the acetabular cup (dislocated) [1]. DDH usually develops in utero or during the neonatal period and affects only one side in 63% of patients [1]. The prevalence of DDH varies between countries (from 1% to 7%) [2], and the female sex reported 2–7 times higher risk [3]. The risk factors include female sex (high levels of estrogen receptors are linked to hyperlaxity) [4]; breech position in utero; firstborn; familiar history; environmental factors (children maintained in wrapped reported lower rates of DDH) [5]. A correct physical examination is necessary to reveal DDH. Positive Ortolani maneuver and limited or asymmetric hip abduction are the most frequent signs [6]. Clinicians need to focus on the asymmetric thigh or gluteal folds and length discrepancy between lower limbs [7], but a negative Galeazzi sign does not exclude the disease [8]. DDH in newborns is suspected from clinical examination and investigated by ultrasonography with Graf classification [9,10].

Rapid diagnosis and treatment are mandatory to avoid complications of DDH (dislocated hip, osteoarthrosis, avascular necrosis of the femoral head and joint stiffness) [11].

The most effective screening protocol for the early detection of DDH is still debated worldwide. Clinical assessment, selective ultrasound or early universal ultrasound screening are adopted as diagnosis methods in several countries [12,13,14]. However, few studies reported the results between countries, making it challenging to redact universal screening guidelines. National health statistics for DDH are attractive for an international audience, as different screening strategies are reported between countries (type of screening, method of ultrasound, mean age at time of screening and diagnosis and subsequent treatment protocols) [12,13,14,15,16]. Sharing national statistics and correlating those to the individual screening systems and treatment protocols, however, could be helpful to compare outcomes for different screening systems internationally.

The objective of this study was to estimate the annual incidence of admissions for DDH in Italian patients from 2001 to 2016, based on the hospitalization reports. In Italy, a selective ultrasound screening method is used to assess an early diagnosis of DDH. Reporting the results of a selective ultrasound screening campaign could be helpful to compare the incidence of DDH hospitalization worldwide. The purpose of this study was to report national results of selective ultrasonography screening to compare them with other countries.

## 2. Materials and Methods

Data of this study were collected from the National Hospital Discharge Reports (SDO) reported at the Italian Ministry of Health regarding the years of this paper (2001–2016). In Italy, the National Health Service (NHS) provides healthcare to all residents. The regional authorities are responsible for organizing and managing the healthcare services delivered through local structures (both public and private accredited providers). Official data on the services provided to residents are collected by hospitals and local healthcare structures, entered into structured data files, and periodically sent to the Ministry of Health. Therefore, the ICD and “procedure codes” are reliable, and the National Hospital Discharge Reports are validated [17,18]. These data were anonymous and reported the patient’s sex, age, days of stay, primary diagnoses, and procedures. Population data from the National Institute for Statistics (ISTAT) for each year were obtained. DDH was defined by the following International Classification of Diseases, Ninth Revision, Clinical Modification (ICD-9-CM) diagnosis codes: 754.30 “Congenital dislocation of hip, unilateral”, 754.31 “Congenital dislocation of hip, bilateral”, 754.32 “Congenital subluxation of hip, unilateral”, 754.33 “Congenital subluxation of hip, bilateral” and 754.35 “Congenital dislocation of one hip with subluxation of other hip”. The ICD-9-CM procedures codes were 77.35 “Other Division of Bone, Femur”, 79.75 “Closed Reduction of Dislocation of Hip”, 79.85 “Open Reduction of Dislocation of Hip” and 83.12 “Adductor Tenotomy Of Hip”. Patients aged between 0 and 14 years were defined as “young” (according to ISTAT) [19]. To avoid underestimating the population which may suffer from DDH, the study was referred only to the young Italian community. Patients with neurological conditions and consequent hip dysplasia were identified using the secondary diagnosis.

### Statistics

The yearly number of DDH, the percentage of males and females, the average age, the average days of hospitalization, primary diagnoses and primary procedures in the whole Italian population were calculated using descriptive statistical analyses. The annual adult population size (achieved from ISTAT, a statutory electronic national population register) were used to calculate the incidence rates. The incidence was based on the size of the entire population of people ≤ 14 years old in Italy. The Statistical Package for Social Sciences (SPSS) version 26 (IBM Corp, Armonk, NY, USA) was used for this data analysis. Figures were created using Excel (Microsoft) software (Microsoft Corporation, Redmond, WA, USA).

## 3. Results

### 3.1. Demographics

**Figure 1:**
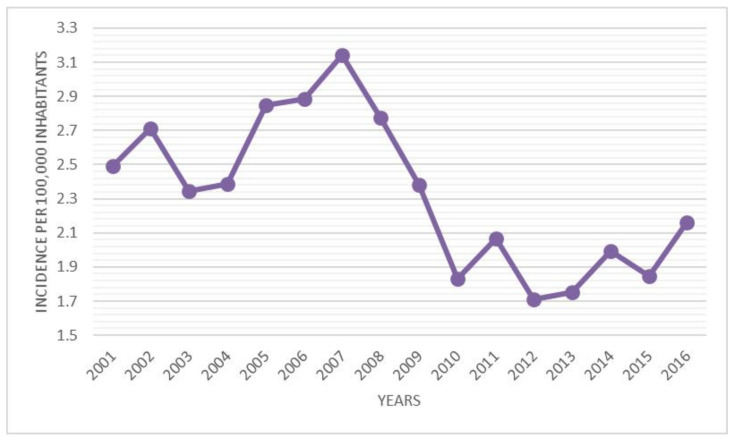
Incidence of DDH hospitalizations from 2001 to 2016 (cases/100,000 inhabitants).

**Figure 2:**
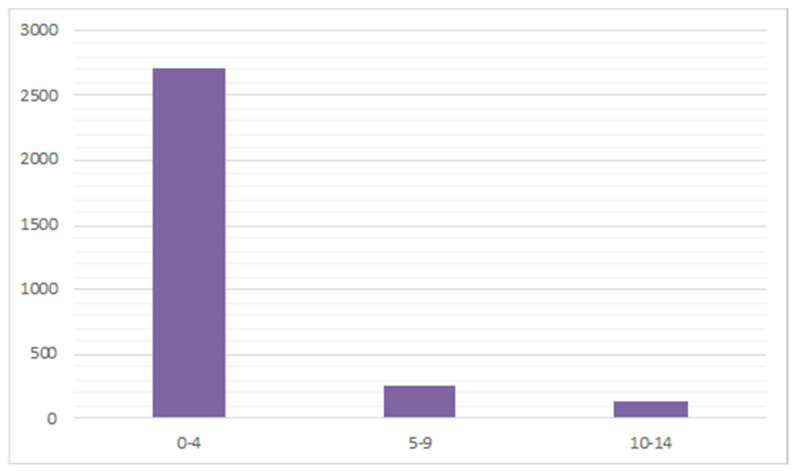
Number of DDH hospitalizations by age group.

**Figure 3:**
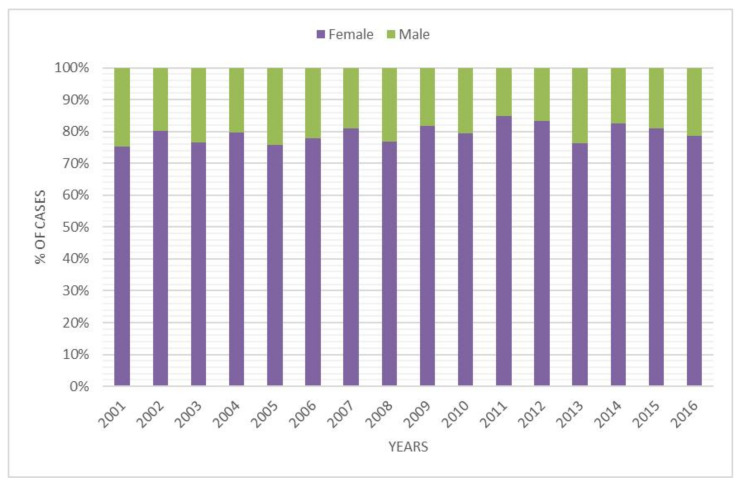
Percentage of DDH hospitalization by years and gender.

**Figure 4:**
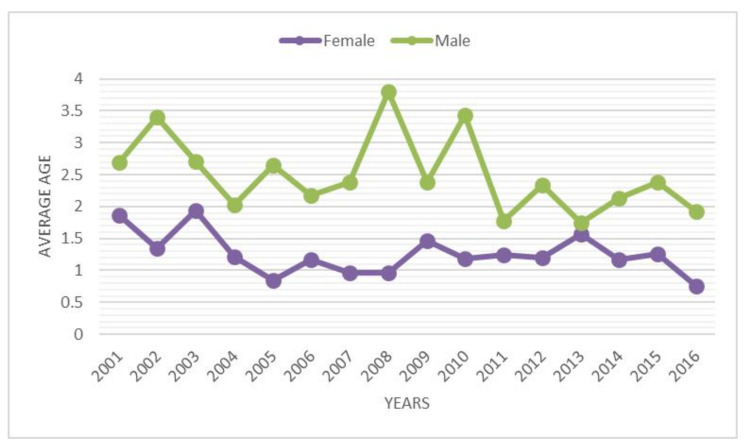
Average age over the years by gender.

During the 16-year study period, 3103 admissions to the hospital for DDH were performed in Italy, representing an incidence of 2.33 procedures for every 100,000 Italian inhabitants 0–14 years old. From 2001 to 2016, the incidence of hospitalizations decreased from 2.49 to 2.16 per 100,000 person-years 0–14 years old (Figure 1). A progressive increase in hospitalizations was recorded from 2003 to 2007. Since 2008, a decrease in hospitalizations has been reported. Over the study period, the highest number of hospitalizations for DDH was found in the 0–4-year age group (Figure 2). In the 0–4 age group, 61.6% of patients underwent “Closed Reduction of Dislocation of Hip”, 21.5% “Open Reduction of Dislocation of Hip”, 10.8% “Adductor Tenotomy Of Hip”. The remaining patients were coded as “Other Division of Bone, Femur”. Females represented the majority of patients undergoing procedures for DDH, both in total and over the years (female 79.3% and male 20.7%) (Figure 3). From 2001 to 2016, the mean age of patients was 1.52 ± 2.96. During the entire period, the average age of males was always higher than females (Figure 4).

### 3.2. Days of Hospitalizations

**Figure 5:**
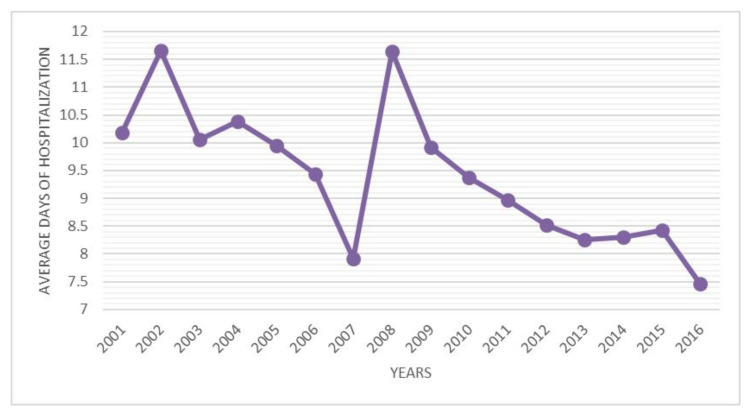
Average days of hospitalization by years.

The average length of hospital stay was 9.5 days (range 0–163 days). The trend of the average number of days of hospitalization was decreasing, with a peak in 2007 (Figure 5). Males had, on average, more days of hospitalization than females (females 9.43 days and males 9.76). Patients aged 0 to 4 had more days of hospitalization on average. Differentiating by sex, males with a higher number of days of hospitalization were between 0 and 4 years old, while women were between 5 and 9 years old.

### 3.3. Procedure Performed and Admission Diagnosis Codes

During the 16-year study period, the main primary diagnoses were “Congenital dislocation of hip, unilateral” (ICD code 75.430; 65%) and “Congenital dislocation of hip, bilateral” (ICD code 75.431; 28.6%). The minor diagnoses were “Congenital subluxation of hip, unilateral” (ICD code 75.432; 3.4%), “Congenital subluxation of hip, bilateral” (ICD code 75.433; 1.8%) and “Congenital dislocation of one hip with subluxation of other hip” (ICD code 75.435; 1.2%).

**Figure 6:**
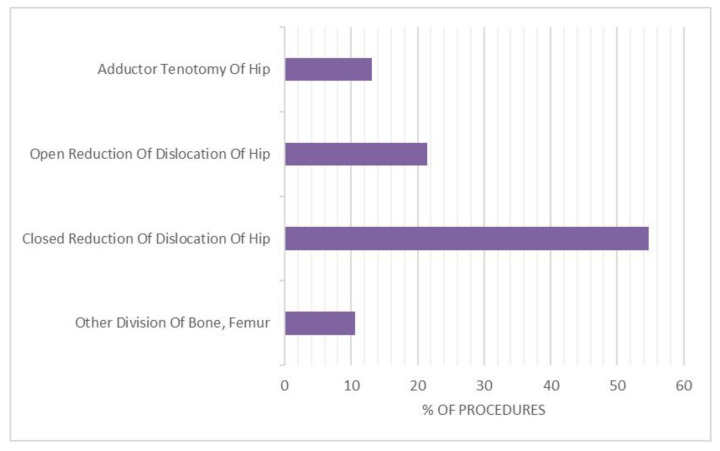
Procedures performed from 2001 to 2016.

**Figure 7:**
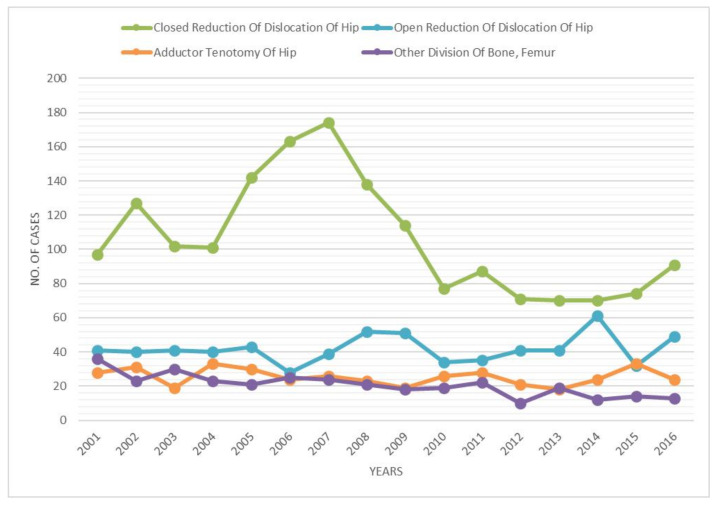
Procedures performed from 2001 to 2016 over the years.

The primary procedures were “Closed Reduction of Dislocation of Hip” (ICD code 79.75; 54.7%), “Open Reduction of Dislocation of Hip” (ICD code 79.85; 21.5%), “Adductor Tenotomy Of Hip” (ICD code 83.12; 13.1%) and “Other Division of Bone, Femur” (ICD code 77.35; 10.6%) (Figure 6). Over the study period, “Closed Reduction of Dislocation of Hip” was prevalent, following by “Open Reduction of Dislocation of Hip” (Figure 7).

**Figure 8:**
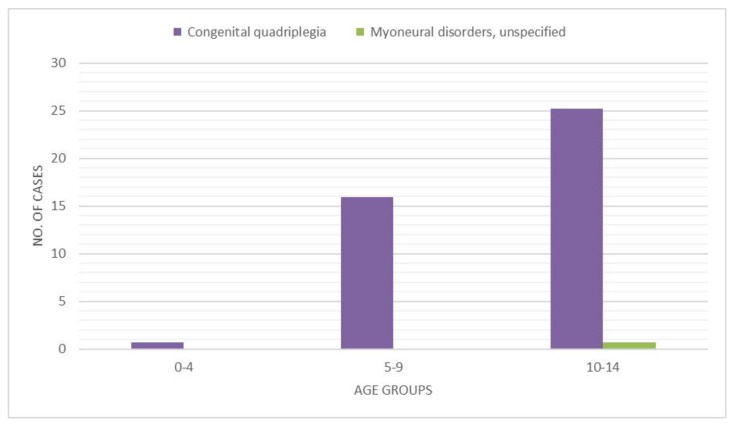
Secondary diagnoses of a neurological nature by age group.

The most significant number of secondary diagnoses of neurological diseases were recorded in patients between 10 and 14 years of age (Figure 7). The secondary diagnoses found were “Congenital quadriplegia” (ICD code 34.32) and “Myoneural disorders, unspecified” (ICD code 35.89). The latter was present in only one patient who was 11 years old (Figure 8).

## 4. Discussion

The objective of this study was to estimate the incidence of hospital admission for DDH in Italian patients from 2001 to 2016. The analysis of SDO records reported a mean incidence of hospital admission for DDH of 2.33 (for every 100,000 inhabitants under 15 years old). A mild decrease in incidence from 2.49 to 2.16 (admission for every 100,000 inhabitants) was reported over the study period. Females between 0 and 4 years old, represent the majority of patients. A mean of 9.5 days of hospitalization was also reported, but it was observed that this value decreased during the years (from 10.18 to 7.46 days). The data reported are in line with other studies [1,5,20].

Early diagnosis and proper treatment are the keys to preventing serious health outcomes for individuals with DDH [21,22]. Otherwise, there are no international guidelines concerning timing, method and type of screening [12,16,23,24]. Therefore, it is mandatory to find an international consensus regarding the screening strategy. In Italy, selective ultrasonography (using Graf classification [10]) is used to assess the presence of DDH. The purpose of this study was to report national results of selective ultrasonography screening to compare them with other countries. Sharing the national statistics could allow the researchers to compare different national screening programs (clinical exams, selective o universal ultrasound). Unfortunately, few studies reported the incidence of DDH hospitalizations worldwide, making it difficult to perform a direct comparison with the present study results [1,12,14,15,16]. Kiung et al. reported that a clinical exam, performed by an experienced clinician, must be obtained before the ultrasound assessment [25], reporting the efficacy of selective ultrasound screening in 2686 infants. Biedermann and colleagues reviewed the literature reporting that the universal ultrasound screening campaign represented the most effective method [13]. However, they also discussed the high costs of this campaign. In 2018, the International Interdisciplinary Consensus Committee on DDH Evaluation (ICODE) tried to achieve consensus on the detection and early treatment of DDH and develop a universal standardized screening program. The ICODE highlighted the effectiveness of a universal ultrasound screening campaign compared to other methods [16]. Treiber and colleagues performed a study on 21,676 newborns between 2006 and 2015 [14]. Additionally, in this study, the superiority of universal ultrasound screening was confirmed. However, further high-quality international studies are required to obtain significant results.

New technologies and screening campaigns reduced the incidence of delayed diagnosis and complication, with a consequent reduction in surgeries for DDH [26]. Moreover, the highest percentage of procedures recorded within the 10–14 age group was performed in patients with neurological diseases. These data confirm the necessity to understand the incidence of this condition in children with cerebral palsy or other musculoskeletal conditions. Further studies that focus on neurological children are required to reach significant conclusions regarding the trend of DDH procedures in this population.

The present study has some limits. It is based on administrative data from different hospitals and macro-regions. We used the International Classification of Diseases 9 (ICD-9) for all the procedures reported. Otherwise, with the ICD-9 used, it was possible to use different codes for the same surgical procedure. The provided database on DDH reported only cases that required hospitalization; therefore, the silent DDHs are not reported. As patients are not recorded with a unique ID number is not possible to distinguish between procedures performed in the same patient or bilateral procedures. This heterogeneity of codification could lead to an underestimation of our results. Lastly, even if the hospital enters the codes, the accuracy of the database is not confirmed. Unfortunately, the healthcare data (including discharge data) are notoriously inaccurate in many countries.

## 5. Conclusions

The incidence of admissions of young patients for DDH in Italy is 2.33 cases/100,000 inhabitants (from 2001 to 2016). DDH requires early diagnosis and treatment; however, no international consensus on screening protocol and treatment is provided in the literature. Epidemiological studies are helpful to understand the national variation of a specific surgical procedure and compare them with other countries. Data provided by different countries could allow researchers to provide international guidelines for DDH screening and treatment.

## Data Availability

The data presented in this study are available on request from the corresponding author. The data are not publicly available due to privacy.
